# Ranking the contribution of behavioral measures comprising oxycodone self-administration to reinstatement of drug-seeking in male and female rats

**DOI:** 10.3389/fnbeh.2022.1035350

**Published:** 2022-11-24

**Authors:** Suman K. Guha, Yanaira Alonso-Caraballo, Gillian S. Driscoll, Jessica A. Babb, Megan Neal, Nicholas J. Constantino, Tania Lintz, Elizabeth Kinard, Elena H. Chartoff

**Affiliations:** ^1^Basic Neuroscience Division, McLean Hospital, Department of Psychiatry, Harvard Medical School, Belmont, MA, United States; ^2^Research Service, VA Boston Healthcare System, Department of Psychiatry, Harvard Medical School, Boston, MA, United States

**Keywords:** escalation, front-loading, female, vulnerability, opioid, relapse, sex difference

## Abstract

**Introduction:**

Rates of relapse to drug use during abstinence are among the highest for opioid use disorder (OUD). In preclinical studies, reinstatement to drug-seeking has been extensively studied as a model of relapse–but the work has been primarily in males. We asked whether biological sex contributes to behaviors comprising self-administration of the prescription opioid oxycodone in rats, and we calculated the relative contribution of these behavioral measures to reinstatement in male and female rats.

**Materials and methods:**

Rats were trained to self-administer oxycodone (8 days, training phase), after which we examined oxycodone self-administration behaviors for an additional 14 days under three conditions in male and female rats: short access (ShA, 1 h/d), long access (LgA, 6 h/d), and saline self-administration. All rats were then tested for cue-induced reinstatement of drug-seeking after a 14-d forced abstinence period. We quantified the # of infusions, front-loading of drug intake, non-reinforced lever pressing, inter-infusion intervals, escalation of intake, and reinstatement responding on the active lever.

**Results:**

Both male and female rats in LgA and ShA conditions escalated oxycodone intake to a similar extent. However, males had higher levels of non-reinforced responding than females under LgA conditions, and females had greater levels of reinstatement responding than males. We then correlated each addiction-related measure listed above with reinstatement responding in males and females and ranked their respective relative contributions. Although the majority of behavioral measures associated with oxycodone self-administration did not show sex differences on their own, when analyzed together using partial least squares regression, their relative contributions to reinstatement were sex-dependent. Front-loading behavior was calculated to have the highest relative contribution to reinstatement in both sexes, with long and short inter-infusion intervals having the second greatest contribution in females and males, respectively.

**Discussion:**

Our results demonstrate sex differences in some oxycodone self-administration measures. More importantly, we demonstrate that a sex- dependent constellation of self-administration behaviors can predict the magnitude of reinstatement, which holds great promise for relapse prevention in people.

## Introduction

Recent attention has focused on the dramatic increase in extensive abuse of prescription opioid drugs and heroin by women, which can lead to opioid use disorder (OUD) and even death through overdose. The 2020 “National Survey on Drug Use and Health: Women” reviewed sex/gender differences in prescription opioid medications and heroin use in the USA from 2015 to 2020 and found that the number of women using heroin is increasing at a faster rate than for men, even though non-medical prescription opioid drug misuse is declining for both sexes (SAMHSA, 2020). A consistent finding has been that the likelihood of substance abuse does not differ between men and women when controlling for access (McHugh et al., 2018). This suggests that vulnerability to initiate drug use is not a major source of sex differences in the addiction cycle. Rather, sex differences in high resolution patterns of opioid use and how these contribute to drug craving and reinstatement are more likely to provide information useful for treatment.

OUD is characterized by chronic cycles of compulsive drug-taking, a loss of control in limiting intake, withdrawal-induced negative affective states, craving, and relapse (Koob, 2020). Clinical evidence points to the negative emotional states of withdrawal combined with high rates of relapse as the major barrier to successful treatment of OUD (Hunt et al., 1971; O’Brien, 2005; Chartoff and Connery, 2014). Considerable research over the past 2–3 decades has identified experimental parameters, neural circuits, and a vast array of molecular substrates underlying both withdrawal-induced aversive states and relapse-like behavior (Chartoff et al., 2006; Russell et al., 2016; Hearing, 2019; Koob, 2020). What remains unknown is how behavioral patterns that manifest during escalation of opioid self-administration directly contribute to the vulnerability and magnitude of reinstatement of drug-seeking after periods of abstinence. Compounding this gap in knowledge is that even less is known about basic behavioral responses to opioid self-administration in females, let alone their ability to predict reinstatement (Becker and Chartoff, 2019). The primary goals of this study were to (1) quantify and compare addiction-relevant behavioral measures observed during oxycodone self-administration in male and female rats to determine if there are sex differences in any individual behavioral measures, and (2) rank the relative contributions of all behavioral measures to post-abstinence reinstatement-responding in male and female rats. Importantly, the question of how each behavioral measures contributes to reinstatement in males and females does not require there to be sex differences in those measures *per se*. Each goal is significant for different reasons. First, the more granular data we can obtain about self-administration behaviors in males and females, the more we can learn about underlying neurobiological mechanisms. Second, the ability to weigh the relative contributions of behaviors displayed during drug self-administration to reinstatement—in males and females separately—suggests this approach may facilitate the ability to accurately predict risk of relapse in men and women with OUD.

To determine how measures and patterns of oxycodone self-administration might be used to predict relapse-like behavior, we used a rat model of OUD that incorporates key features of OUD in people, including escalation of drug intake when availability of drug is extended (i.e., long-access, LgA)(Ahmed et al., 2000; Belin-Rauscent et al., 2016; Towers et al., 2019), followed by 14 days of forced abstinence and a subsequent reinstatement test in which rats underwent a standard self-administration session with the same cues and contexts previously paired with oxycodone, but presses on the active lever did not deliver drug (Zhou et al., 2009; Venniro et al., 2016). We focused on self-administration behavioral measures relevant to OUD in people for analysis of their predictive ability: oxycodone infusions, escalation of intake, front-loading, and inter-infusion intervals. The concept of using addiction-like behaviors to generate individualized measures that can predict vulnerability to reinstatement or relapse to SUD itself is not new (Kruzich et al., 1999; Belin et al., 2009; O’Neal et al., 2020; Panlilio et al., 2020), but has not, to our knowledge, been examined in both males and females or within the context of abused prescription painkillers like oxycodone.

## Materials and methods

### Animals

Adult female (*N* = 52) and male (*N* = 51) Sprague-Dawley rats (Charles River Laboratory, Wilmington, MA) were used. Body weights upon arrival were 200–225 g (female) and 250–275 g (male). Upon arrival, rats were group housed (4 rats/cage) and were acclimated for 1 week in a 12-h light-/dark-cycle (7:00 am/7:00 pm lights on/off) with food and water available *ad libitum*. Following surgeries, all rats remained singly housed. All experiments were conducted during the light phase. All guidelines recommended by the Institutional Animal Care and Use Committee of McLean Hospital and by the National Institutes of Health guidelines for the care and use of laboratory animals were followed.

**FIGURE 1:**
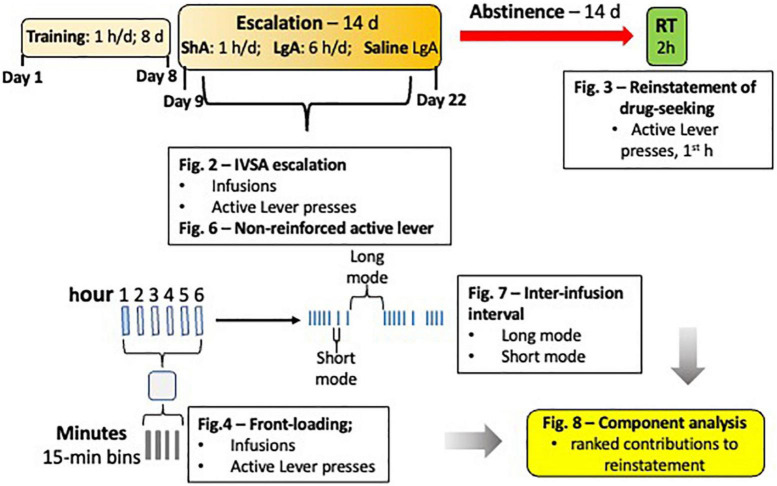
Experimental Schematic. The IVSA paradigm is depicted with the orange boxes, forced abstinence with the red arrow, and the reinstatement test with the green box. A blow up of one example day is shown below the overall schematic, with an example IVSA session represented as long access (LgA), with 6 bars representing 6 h [First blue bar indicates a short access (ShA) session)]. Below that, 1 h of one session is represented by 4 smaller bars representing 15-min bins. To the right of the blow up of a representative session is an example of infusion patterns, with blue ticks representing infusions. Text boxes represent the behavioral measures analyzed for sex differences and used in regression analyses to determine contribution to reinstatement responding and ability to predict magnitude of reinstatement.

The data analyzed for this study is a combination of data generated by 3 different investigators in the lab for their individual projects. All 3 experiments were run in the same operant chambers and used the same IVSA paradigm shown in Figure 1: an 8-d training phase (1-h/d), a 14-d escalation phase (either 1 or 6 h/d), a 14-d forced abstinence phase, and a cue-induced reinstatement test. The first experiment included 26 female and 26 male rats. Approximately 6 weeks prior to catheterization surgery for oxycodone self-administration, intracranial self-stimulation (ICSS) stimulating electrodes were surgically implanted in the medial forebrain bundle at the level of the lateral hypothalamus, as in Carlezon and Chartoff (2007) and Ebner et al. (2010). The IVSA paradigm used in rats from Experiment 1 was identical to the other groups and is described below in the Methods. See Supplementary methods for details of the ICSS portion of Experiment 1. The second experiment included 21 female (saline, *N* = 10; oxycodone, *N* = 13) and 22 male (saline, *N* = 9; oxycodone, *N* = 11) rats that underwent the IVSA paradigm described below. The third experiment included 3 male and 5 female rats that underwent the IVSA paradigm described below. For simplicity, data from the rats of Experiments 2 and 3 have been combined.

An important question is whether the presence of the ICSS electrode and/or ICSS behavior altered self-administration behavior such that combining data from these experimental cohorts is not feasible. As such, examples of IVSA data from Experiment 1 (IVSA + ICSS) and Experiment 2 (IVSA alone) are compared in Supplementary Figure 1. We show no significant differences between experimental groups for self-administration infusions, front-loading behavior, and reinstatement responding.

### Intravenous catheter implantation

All rats were implanted with chronic indwelling silastic intravenous (i.v.) jugular vein catheters (0.51 mm internal diameter; SAI Infusion Technologies, IL, USA) as described (Mavrikaki et al., 2017, 2020). Briefly, rats were anesthetized with a ketamine/xylazine mixture (ketamine, 80 mg/kg; xylazine 8 mg/kg, IP), and catheters were implanted into the right jugular vein, secured to the vein with non-absorbable sutures, and the tubing passed subcutaneously to exit dorsally from the rat’s back. After surgery, rats were injected once with ketoprofen (5 mg/kg, SC) to reduce pain. For maintenance, catheters were flushed daily with 0.2 ml of heparinized saline (30 USP units/ml) and once a week with 0.2 ml gentamicin (10 mg/ml). Catheter patency was tested at least once per week by i.v. infusion of 150–200-μl methohexital sodium (2 mg in 200 μl for the males and 1.5 mg in 150 μl for the females, i.v.) and assessing loss of righting reflex. If the latency to loss of righting was greater than 10 s, the catheter was not considered patent, and the rat was not tested.

### Intravenous self-administration

Intravenous self-administration (IVSA) was performed during the light phase in operant conditioning chambers (11.625” L 9.78” W 10.75” H; Med Associates, St. Albans, VT, USA) with grid floors and contained in sound- and light-attenuating outer chambers (ENV-007CT; Med Associates). A syringe pump located outside each chamber was connected to a swivel within each chamber to permit IV drug infusions. After 1-week recovery from catheter implantation surgery, rats were trained to self-administer oxycodone (provided by the NIDA Drug Supply Program) at 60 μg/kg per infusion on a fixed ratio 1 (FR1) schedule of reinforcement. For training, 1-h sessions [i.e., short access (ShA)] were conducted once per day (M-F) for 8 d, as in Schlosburg et al. (2013) and Mavrikaki et al. (2019). Rats then entered the escalation phase, comprising 14 d of oxycodone self-administration (M-F) under either ShA (1 h/day) or long access (LgA; 6 h/day) conditions. A subset of rats underwent this IVSA paradigm with saline instead of oxycodone (*N* = 10 females, 9 males). Each self-administration trial began with the chamber house light turning on and the extension of two lever manipulanda signaling drug availability. A press on the active lever resulted in illumination of a cue light over the active lever and the house light turning off. Coincident with presentation of cues, a syringe pump delivered 100 μl of oxycodone (60 μg/kg per infusion) lasting 4 s. Following the 4-s infusion was a 6-s timeout period during which any press on the active lever had no consequence. Following the full 10-s cycle, the cue light turned off and the house light turned on to again signal drug availability. Presses on the inactive lever were recorded but had no consequences. We recorded and analyzed total infusions, active lever presses (those that led to a drug infusion and those that occurred during a timeout), and inactive lever presses.

### Cue-induced reinstatement test

After the last IVSA day, rats were returned to their home cages in the vivarium for 14-d of forced abstinence; a design based on studies using heroin (Zhou et al., 2009; Fanous et al., 2012). After the abstinence period, rats were placed in the same operant chambers in which they had previously self-administered oxycodone. A 2-h reinstatement test was conducted, with conditions identical to those during self-administration except a press on the active lever did not result in drug (oxycodone or saline) infusion. Both Zhou et al. (2009) and Fanous et al. (2012) showed that cue-induced reinstatement responding on the active lever is significantly higher after 14 days compared to 1 day of forced abstinence, consistent with time-dependent increases in drug-seeking; i.e., incubation of craving (Shalev et al., 2001). In our study, we only conduct one cue-induced reinstatement test (after 14-d abstinence), which means we are not directly measuring the incubation phenomenon. As such, we compare reinstatement responding in males and females among the three treatment groups (LgA, ShA, and saline self-administration).

### Statistical analyses

Behavioral data was collected using automated procedures (MedPC-IV, MedAssociates) and processed using custom written scripts in R (R Core Team, 2019). Further preparation of data and statistical analysis was done with R and GraphPad Prism 9 (GraphPad Software, San Diego, CA, USA). For treatment by sex by time comparisons, 3-way ANOVAs with repeated measures on time were used. In the event of significant interactions or main effects, data were collapsed into the relevant two factors, and 2-way ANOVAs were performed. For sex by time comparisons, 2-way ANOVAs with repeated measures on time were used. In the event of significant interactions or main effects, Sphericity was not assumed, so the Geisser-Greenhouse correction was used. To understand the difference between various time-points, multiple comparison tests were performed.

One goal of these studies was to identify key behaviors measured during the escalation phase of oxycodone self-administration that correlate with reinstatement-responding after prolonged abstinence from self-administration. Various measures of drug self-administration behavior have been related to addiction severity (O’Neal et al., 2020). We tested for correlations between reinstatement responding and 6 measures of oxycodone self-administration behavior. The six behavioral measures were all taken from the escalation phase of oxycodone self-administration (days 9–22), and are as follows *(1) infusion score*, the average total number of infusions; *(2) front-loading*, the average number of infusions during the first 15 mins of the self-administration day; *(3) Active (no reward)*, the average number of non-reinforced active lever presses normalized to the number of infusions; *(4) escalation score*, the difference in oxycodone infusions between day 9 and 22); *(5 and 6) inter-infusion interval (long and short)*, the average of the mode of time-difference (s) between two successive bursts of oxycodone infusions. Inter-infusion intervals showed a bimodal distribution, suggesting there were two predominant time-differences (long and short). First univariate (Pearson’s) correlation analysis was performed to determine if each of these behavioral measures were significantly associated with active lever presses during the reinstatement test. Additionally, Pearson’s correlation between these measures was performed to determine whether the measures themselves were correlated with each other, also known as collinearity. To understand how measures of addiction-like behavior (that are correlated with each other) differentially affect reinstatement of drug-seeking in females and males, multivariate (partial least squares) regression analysis was used (Kuhn and Johnson, 2018) and variable importance in projection was measured. Variable importance in projection is a measure calculated using the weighted sums of the absolute regression coefficients (Kuhn, 2008). See Figure 1 for an Experimental Schematic.

## Results

### Male and female rats escalate oxycodone intake under both long access and short access conditions

**FIGURE 2:**
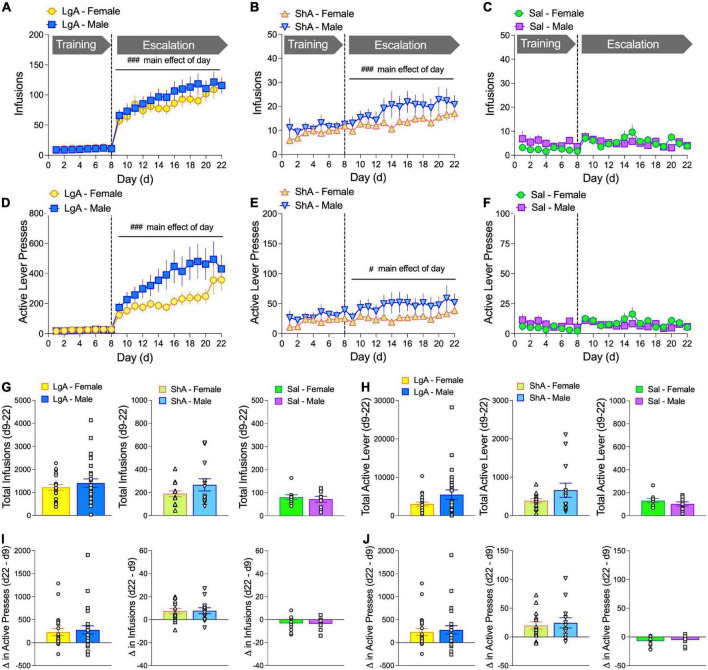
Female and male rats escalate oxycodone self-administration under both long access (LgA, 6 h) and short access (ShA, 1 h) conditions. Oxycodone self-administration behavior is shown as # of infusions/day **(A–C)** and # of active lever presses/day **(D–F)** for male and female rats across the 22 days of the self-administration paradigm. Groups included rats that self-administered oxycodone under LgA conditions **(A,D)** or under ShA conditions **(B,E)** during the escalation phase (days 9–22) and rats that self-administered saline **(C,F)**. Under both LgA and ShA, but not saline, conditions, there was a main effect of Day. There were no sex differences in total infusions **(G)** or active lever presses **(H)** during the escalation phase (d9–22). There were no sex differences in the change in number of infusions **(I)** or active lever presses **(J)** from escalation day 9 to escalation day 22, although the net change for LgA and ShA, but not saline, rats was >0, indicating escalation of intake (see Supplementary Figure 2). ^#^*p* < 0.05, ^###^*p* < 0.001, main effect of Day. *N*: LgA (22 females, 26 males); ShA (15 females, 13 males); Sal (10 females, 9 males).

Currently, there is relatively little information comparing oxycodone self-administration behaviors between male and female rats. Here we report data on several behavioral measures associated with oxycodone self-administration during an escalation phase in which male and female rats have either long access (LgA) or short access (ShA) to drug, or in which male and female rats self-administer saline. It has typically been found that laboratory animals will escalate drug intake when allowed LgA, but not ShA, to drug (Ahmed and Koob, 1998; Ahmed et al., 2000). Escalation of heroin intake has been reported in male and female mice (Towers et al., 2019), suggesting similar effects will be observed with oxycodone. In our study, rats underwent an 8-d training phase, in which rats could self-administer oxycodone (60 mg/kg/infusion) for 1 h/day (1 h/d, M-F), followed by a 14-d escalation phase in which rats could self-administer oxycodone under ShA (1-h/d) or LgA (6-h/d) conditions (M-F). A separate group of rats self-administered saline under LgA conditions. The number of infusions and active lever presses increased over the course of the escalation phase (days 9–22) under both LgA and ShA conditions. Main effects of Day were found for Infusions (LgA: *F*_(4.53, 240.1)_ = 10.20, *p* < 0.0001, Figure 2A; ShA: *F*_(4.50, 115.6)_ = 5.81, *p* < 0.0001, Figure 2B) and Active Lever presses (LgA: *F*_(2.79, 148.0)_ = 8.96, *p* < 0.0001, Figure 2D; ShA: *F*_(3.88, 101)_ = 2.618, *p* = 0.041, Figure 2E). Rats that self-administered saline did not show an increase in responding throughout the escalation phase (Figures 2C,F). No Sex × Day interactions or main effects of Sex were observed under either LgA or ShA conditions. To control for possible non-specific motor effects, which could indicate a lack or reduction of learning the drug-lever pairing—we found no significant changes in inactive lever presses over time or by sex (Supplementary Figure 2).

Despite the lack of sex effects in oxycodone self-administration over the course of the escalation phase, the number of infusions and active lever presses was visually higher in males than females. To determine if there was a sex difference in overall drug intake or lever pressing, we compared total infusions and active lever presses in males and females during the escalation phase (days 9–22). Unpaired *t*-tests for all each (Figures 2G,H) showed no significant sex effects. In addition, we calculated the difference in oxycodone infusions (Figure 2I) and active lever presses (Figure 2J) between escalation days 9 and 22 as a measure of escalation and compared this escalation score between males and females. Unpaired *t*-tests for each treatment condition revealed no significant sex differences.

To specifically determine if rats escalated their drug intake, we compared the number of infusions and active lever presses in males and females on day 22 to those on day 9 for each treatment condition (LgA, ShA, saline). Two-way ANOVA (Sex × Day) with repeated measures on Day showed no Sex × Day interactions or main effects of Sex under any condition. However, there were main effects of Day for infusions and active lever presses under all conditions. Bonferroni’s multiple comparison tests showed that both males and females had significantly higher oxycodone infusions and active lever presses on escalation day 22 (ESC d22) compared to ESC d9 in LgA and ShA rats (Supplementary Figure 3). Main effect of Day for Infusions [LgA: *F*_(1, 54)_ = 29.72, *p* < 0.0001, **(A)**; ShA: *F*_(1, 26)_ = 20.43, *p* = 0.0001, **(B)**; Sal: *F*_(1, 17)_ = 6.12, *p* = 0.024, **(C)**] and main effect of Day for Active lever presses [LgA: *F*_(1, 49)_ = 23.56, *p* < 0.0001, **(D)**; ShA: *F*_(1, 26)_ = 17.64, *p* = 0.0003, **(E)**; Sal: *F*_(1, 17)_ = 9.56, *p* = 0.007, **(F)**].

### Reinstatement responding is higher in female compared to male rats after 14-d of forced abstinence from oxycodone self-administration

A major goal of these studies was to determine if behavioral measures observed during oxycodone self-administration—relevant to DSM-5 criteria for OUD (American Psychiatric Association, 2013)—can be ranked in importance to their contribution to reinstatement of drug-seeking after a period of abstinence. As such, our experimental design included a 14-d forced abstinence period after the last self-administration day (day 22) followed by a cue-induced reinstatement test. This design was intended to approximate a common experience in people with substance use disorder in which periods of heavy drug use are followed by periods of abstinence (often forced, e.g., incarceration) followed again by reinstatement to drug-taking (Reiner et al., 2018).

**FIGURE 3:**
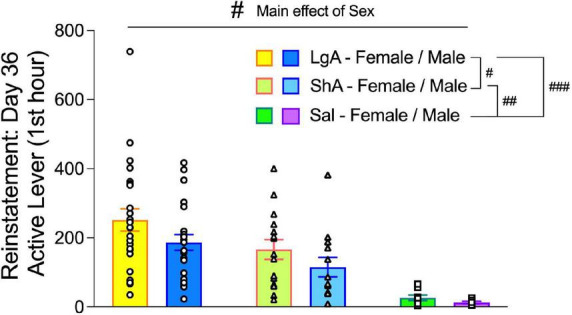
Reinstatement responding after 14 days of forced abstinence: effects of sex and prior self-administration condition. Data are shown as the average number (±SEM) of active lever presses during the first hour of the Reinstatement Test in male and female rats that had self-administered oxycodone in the escalation phase under LgA or ShA conditions or self-administered saline (Sal). Across treatment groups, females had higher levels of reinstatement responding than males (main effect of Sex). Further, treatment group dictated the level of reinstatement responding, with LgA > ShA > Sal (main effect of Treatment). ^#^*p* < 0.05, ^##^*p* < 0.01, ^###^*p* < 0.001, main effects of sex and treatment, as indicated by brackets. *N*: LgA (19 females, 20 males); ShA (15 females, 13 males); Sal (8 females, 6 males).

Importantly, there is a large body of literature showing that craving for drug can “incubate” (increase), resulting in different levels of reinstatement in a time- and drug-dependent manner (Reiner et al., 2018). In our study, all rats experienced 14 days of abstinence, which did not allow measurement of the incubation of craving. As such, we compared the magnitude of reinstatement responding between males and females among the three treatment conditions (LgA, ShA, Sal). Active lever pressing during the reinstatement test was higher in females compared to males across treatment groups (Figure 3), which depended on a main effect of Sex (*F*_(1, 85)_ = 4.57, *p* = 0.035). Reinstatement responding was highest in rats that had self-administered oxycodone under LgA conditions, which depended on a main effect of Treatment (*F*_(2, 85)_ = 17.06, *p* < 0.0001).

### Female and male rats exhibit front-loading self-administration behavior

It is well established that drug self-administration typically starts with a “front-loading phase,” in which an animal makes several lever presses in rapid succession to quickly bring blood/brain drug levels to a concentration that is reinforcing. This is followed by a “maintenance phase” in which drug self-administration occurs at more regular intervals thought to maintain sufficient drug levels to sustain the desired hedonic set point (Wise and Bozarth, 1987; Gerber and Wise, 1989). However, repeated opioid use usually leads to tolerance and dependence (Wise and Koob, 2014); conditions in which progressively increasing intake is required to front-load and maintain drug effectiveness (Ahmed and Koob, 1998, 2005).

**FIGURE 4:**
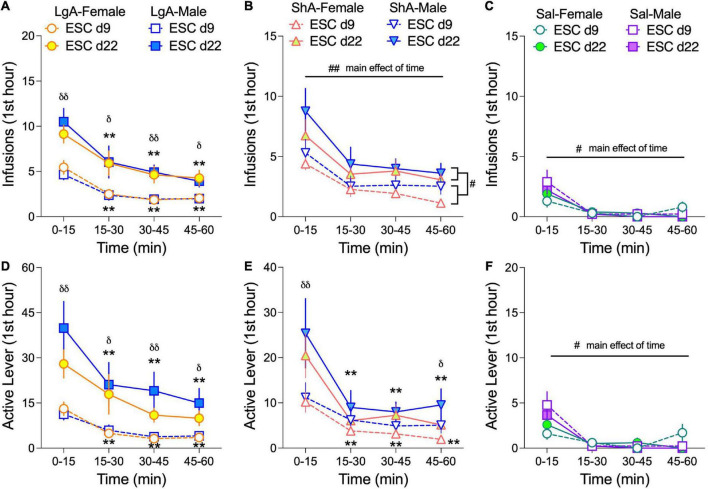
Female and male rats front-load oxycodone intake. Data are shown as the average number (±SEM) of infusions **(A–C)** and active lever presses **(D–F)** in 15-min bins from the first hour of escalation day 9 (ESC d9) and day 22 (ESC d22) in male and female rats. For each treatment condition: LgA **(A,D)**, ShA **(B,E)**, and Sal **(C,F)**, 3-way ANOVAs (Sex × ESC day × Time) were calculated. For oxycodone self-administration under LgA and ShA conditions, rats self-administered more oxycodone in the first 15-min bin compared to the last 15-min bin (i.e., front-loading), and overall oxycodone intake was greater on ESC d22 compared to ESC d9. This was determined through main effects or interactions of ESC day and Time. ^#^*p* < 0.05, ^##^*p* < 0.01, main effect of Time; **p < 0.01, Dunnett’s multiple comparisons to the 0–15 min bin; ^δ^*p* < 0.05, ^δδ^*p* < 0.01 Bonferroni’s multiple comparisons of ESC d9 to ESC d22 per time bin. N: LgA (19 females, 20 males); ShA (15 females, 13 males); Sal (8 females, 6 males).

To determine if male and female rats exhibit front-loading behavior during the escalation phase of self-administration and if front-loading changes from the beginning to the end of escalation (d9 vs d22), we analyzed the first hour of self-administration data in 15-min bins on escalation day 9 (ESC d9) and ESC d22 (Figure 4). We found that oxycodone (or saline) infusions and active lever presses were greatest in the first 15 min of the self-administration sessions on both ESC d9 and ESC d22 under every treatment condition (LgA, ShA, and Sal). This robust evidence for front-loading depended on either Day × Time interactions (Figures 4A,D,E) or main effects of Time (Figures 4B,C,F) in the 3-way ANOVAs (Sex × Day × Time) conducted for each treatment condition. An example of a Day × Time interaction is found in active lever pressing in LgA rats (Figure 4D): (*F*_(2.17, 99.68)_ = 3.83, *p* = 0.022), and an example of a main effect of time is found in infusions in Sal rats (Figure 4C): (*F*_(3.0, 51.0)_ = 20.46, *p* < 0.0001). The fact that rats self-administering saline had higher infusions (Figure 4C) and active lever presses (Figure 4F) in the first 15 min of the sessions suggests that a portion of front-loading behavior can be attributed to locomotor stimulation from placing the rats in the operant chambers each day. The 3-way ANOVAs for this front-loading data also revealed that oxycodone, but not saline, self-administration behavior was higher across the first hour of ESC d22 compared to ESC d9 in LgA and ShA rats, consistent with development of tolerance. There were no effects of sex on front-loading behavior.

### Temporal features of long access oxycodone self-administration sessions at the beginning and end of the escalation phase

**FIGURE 5:**
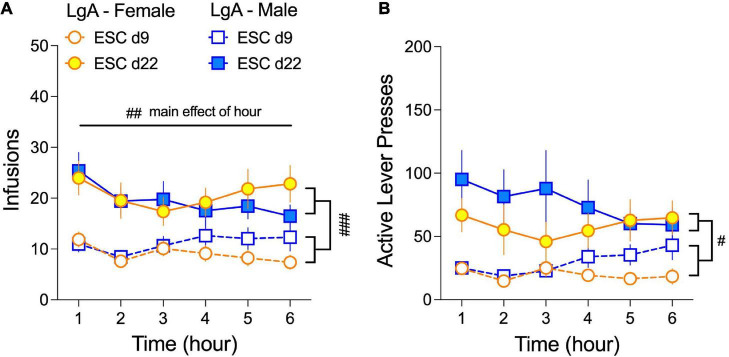
Female and male rats self-administer oxycodone throughout the 6-h LgA sessions during the escalation phase. The average number (±SEM) of oxycodone infusions **(A)** and active lever presses **(B)** for each of the 6 h of escalation days 9 (ESC d9) and 22 (ESC d22) are plotted for males and females. Infusions and active lever presses are higher for both sexes on ESC d22 compared to ESC d9 (main effect of Day), although there is an overall, yet modest, decrease in responding across the 6-h (main effect of Hour). No sex differences were observed. ^#^*p* < 0.05, ^##^*p* < 0.01, ^###^*p* < 0.001, main effects of day and hour, as indicated. N: LgA (19 females, 20 males); ShA (15 females, 13 males); Sal (8 females, 6 males).

Male and female rats do not differ in the number of oxycodone infusions or active lever presses during LgA sessions of the escalation phase (d9–22; Figures 2A,D). However, it is not known whether self-administration behavior fluctuates within a 6-h session or between early (d9) and late (d22) 6-h sessions in male and female rats. As such, we quantified oxycodone infusions (Figure 5A) and active lever presses (Figure 5B) for each hour of the 6-h IVSA sessions in LgA rats on escalation days 9 (ESC d9) and ESC d22. We found that rats exhibited front-loading behavior at the hourly level, as the number of infusions was highest in the first hour of the 6-h session on both ESC d9 and ESC d22. This depended on a main effect of Hour in the 3-way ANOVA (Sex × Day × Hour) for infusions: (*F*_(5.0, 230)_ = 3.62, *p* = 0.004). Front-loading was not observed with active lever presses, and no significant effects of sex were found in any condition. Finally, hourly infusions and active lever pressing were higher across the 6-h sessions on ESC d9 compared to ESC d22, which depended on main effects of Day in the 3-way ANOVAs for infusions (*F*_(0.76, 34.94)_ = 29.29, *p* < 0.001) and active lever presses (*F*_(0.45, 20.61)_ = 17.88, *p* = 0.003).

### Males have higher levels of non-reinforced active lever presses compared to females during the escalation phase of long access oxycodone self-administration

**FIGURE 6:**
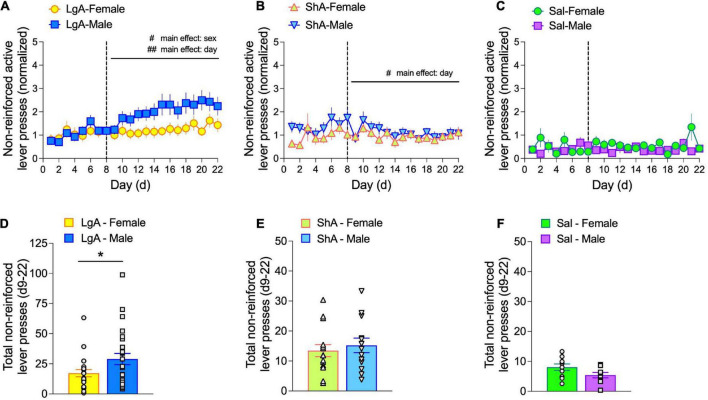
Males exhibit higher levels of non-reinforced active lever pressing than females under LgA self-administration conditions. Non-reinforced active lever pressing is expressed as: [(non-reinforced active lever presses—infusions)/infusions]. Data are shown as non-reinforced active lever pressing per self-administration day (d1–22) for LgA **(A)**, ShA **(B)**, and Sal **(C)** escalation phase treatment groups and as the total of non-reinforced active lever pressing during the escalation phase (d9–22) for LgA **(D)**, ShA **(E)**, and Sal **(F)** escalation phase treatment groups. Under LgA conditions, non-reinforced active lever pressing depended on main effects of Sex and Day **(A)**, with males showing significantly higher responses than females **(D)**; unpaired *t*-test). ^#^*p* < 0.05, ^##^*p* < 0.01, main effects of sex and day, as indicated; *p < 0.05 unpaired *t*-test. *N*: LgA (22 females, 26 males); ShA (15 females, 13 males); Sal (10 females, 9 males).

Each active lever press that leads to drug delivery triggers a 4-s infusion (100 μl) followed by a 6-s time out period. Any active lever presses that occur during the total 10 s after an infusion-triggering active lever press are not reinforced with drug delivery. To quantify continued lever pressing during these non-reinforced periods, we summed the normalized non-reinforced lever presses for each day of IVSA using the formula [(active press—infusion)/infusion]. The more non-reinforced lever presses per session, the higher the value of this output. A value of 1 means there were at least twice as many active lever presses as infusions, whereas a value between 0 and 1 means the number of active lever presses was less than twice the number of infusions. We observed that both male and female rats self-administering oxycodone in 1-h sessions—during the training or escalation phases—had a normalized, non-reinforced active lever press value of ∼1 (Figures 6A,B). This suggests that rats typically press the active lever about twice as often as they receive oxycodone infusions. In rats self-administering saline; however, we observed a normalized, non-reinforced active lever press value of ∼0.5 (Figure 6C). Taken together, these observations are consistent with a higher motivation to seek and acquire reinforcing stimuli (e.g., oxycodone) but not non-reinforcing stimuli (e.g., saline).

During the escalation phase under LgA, but not ShA or saline, conditions, a sex difference emerged such that normalized, non-reinforced active lever presses were higher in males compared to females (Figure 6A). This depended on a main effect of Sex (*F*_(1.0, 46)_ = 4.15, *p* = 0.047). A main effect of Day was also found (*F*_(4.44, 204.2)_ = 4.83, *p* = 0.0006), but pairwise comparisons revealed no significant differences among escalation days. The sex difference in normalized, non-reinforced active lever presses was also observed when these data were summed over the escalation phase in LgA rats (Figure 6D), but not ShA or Sal rats (Figures 6E,F, respectively).

### The time (interval) between bursts of oxycodone infusions decreases as escalation of intake increases in male and female long access rats

**FIGURE 7:**
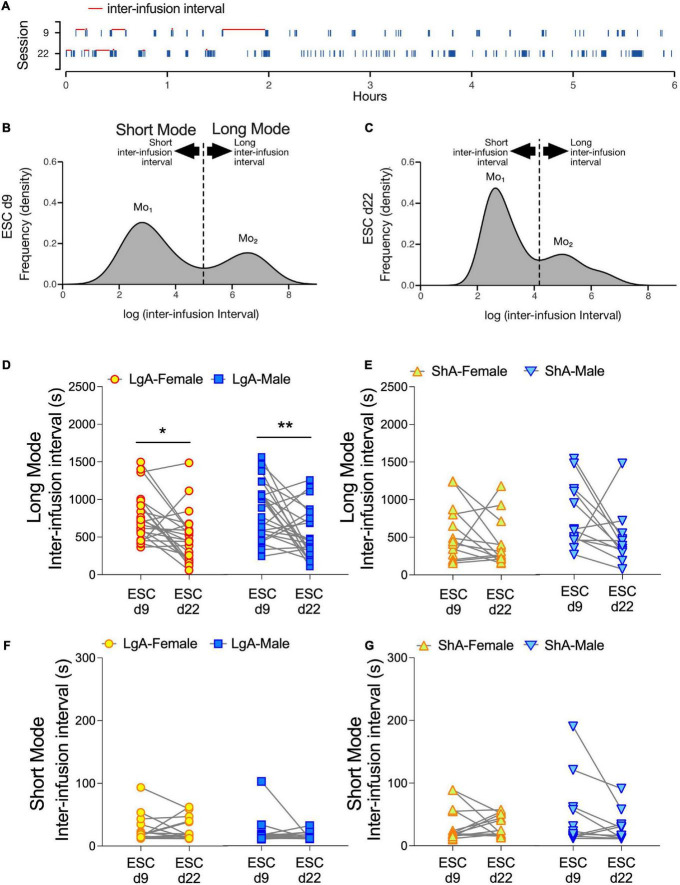
Latency between bursts of oxycodone infusions decreases in females and males under LgA, but not ShA, self-administration conditions. Schematics illustrating methods to calculate interval latencies between oxycodone infusions are generated from representative data and shown in **(A–C)**. In panel **(A)**, raster plots show oxycodone infusions (blue ticks) from the escalation days 9 and 22 from a LgA rat. All inter-infusion intervals (s) are quantified example intervals demarcated with red lines **(A)**. Frequency distributions of log-transformed inter-infusion intervals across the 6-h self-administration days reveal bimodal distributions that consist of a population of long (long mode), and a population of short (short mode), intervals **(B,C)**. Mo_1_ represents the inter-infusion interval in the short mode with the highest frequency of occurrence, and Mo_2_ represents the inter-infusion interval in the long mode with the highest frequency of occurrence. Data in panels **(D–G)** are dot and line plots representing average inter-infusion intervals for the long mode **(D,E)** and short mode **(F,G)** on escalation days 9 (ESC d9) and 22 (ESC d22) for each rat under LgA **(D,F)** and ShA **(E,G)** conditions. 2-way ANOVAs (Sex × ESC day) show a main effect of ESC day and a significant decrease in Long Mode inter-infusion intervals in both males and females under LgA conditions on ESC d22 compared to ESC d9 **(D)**. *p < 0.05, **p < 0.01, Bonferroni’s multiple comparisons of ESC d9 to ESC d22 per sex. LgA (22 females, 26 males); ShA (15 females, 13 males).

Development of a burst-like pattern of drug self-administration has been associated with addiction-like behavior in cocaine self-administration studies (Belin et al., 2009). Historically, burst-like patterns are defined by a minimum number of drug infusions over a specified time-window. However, this approach does not account for the variable pattern of infusions during a burst nor the dynamic nature of burst-like patterns over time. To address this, we used inter-infusion intervals (s) as a proxy for drug self-administration patterns. An evenly spaced drug self-administration pattern would have a normal/gaussian distribution of inter-infusion intervals. A burst-like drug self-administration pattern would have a bimodal distribution (Figures 7A–C). Based on empirical observation of oxycodone infusion histograms from self-administration sessions (e.g., Figure 7A), the larger mode (long-mode) typically indicates time elapsed between two burst-like bouts of oxycodone self-administration, whereas the smaller mode (short-mode) typically indicates time elapsed between two successive infusions within a burst-like bout. Both LgA and ShA rats undergoing oxycodone self-administration showed this bimodal distribution of inter-infusion intervals. In male and female LgA rats, the long-mode intervals decreased as drug escalation increased from days 9 to 22 (Figure 7D). This was supported by a main effect of Day (*F*_(1.0, 43)_ = 16.88, *p* = 0.002) and Bonferroni’s multiple comparison tests. In contrast, the short-mode intervals remained unchanged (Figure 7F). Together, these findings suggest that the nature of the bursts remains the same, but their frequency increases.

### Sex differences in the relative contributions of oxycodone self-administration behaviors to reinstatement responding

**FIGURE 8:**
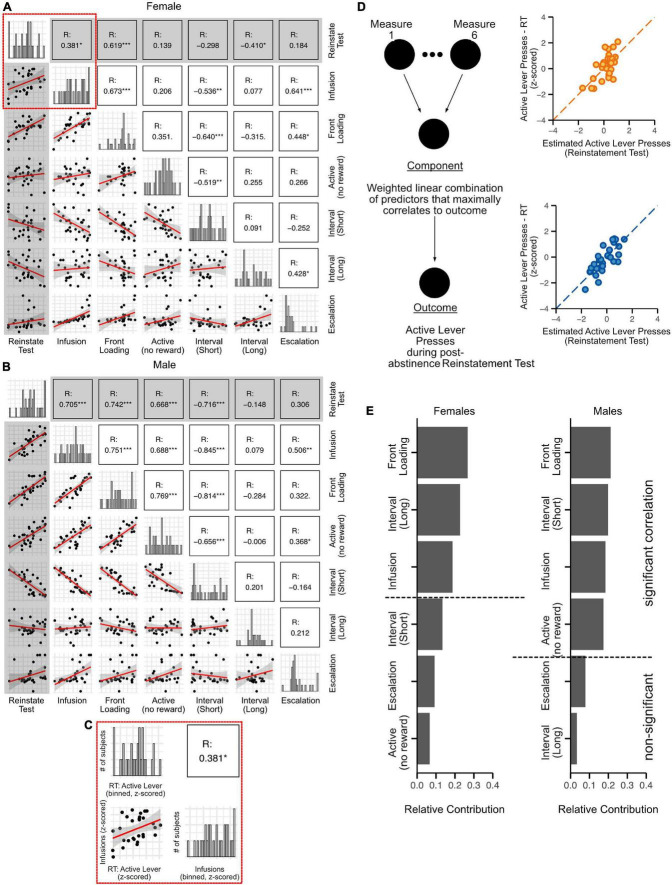
Relative contribution of behavioral measures associated with oxycodone self-administration to post-abstinence reinstatement responding. The 6 behavioral measures quantified in the previous figures [Infusions, front-loading, non-reinforced active lever pressing, Inter-infusion intervals (short and long modes), Escalation] were segregated by sex, *z*-scored, and correlated with reinstatement responding (Pearson’s). For both females **(A)** and males **(B)**, histograms across the diagonal represent *z*-scored distributions of each measure. The inset **(C)** provides enlarged visualization of each component of the matrix. In the histograms, the X-axis represents the *z*-scored measures, while the Y-axis represents the number of rats. The correlation plot in panel **(C)** shows the relationship between *z*-scored active lever presses in the reinstatement test (RT: active lever) and *z*-scored oxycodone infusions (infusion). The correlation matrix (below the diagonal of histograms) represents the cross-correlation between all the measures, while the matrix on top shows the correlation coefficients (R). Significant correlations are indicated with asterisks. Gray highlighted squares show comparisons of reinstatement test responding with each behavioral measure: correlation plots are in the gray-shaded column and correlation coefficients (R) are in the gray-shaded row. **(D)** Schematic showing the partial least square regression model used to determine the role of each measure to reinstatement responding. The dot plots in panel **(D)** (orange circles, females and blue circles, males) were generated with the partial least square regression model and demonstrate a tight fit between observed reinstatement responses vs predicted ones. Bar plots were generated indicating the relative contribution of each behavioral measure to reinstatement test responding in female and male rats **(E)**. N: 30 females, 29 males.

An important goal of this study was to determine how the various measures of addiction-like behavior reported here contribute to reinstatement responding, with the relative contributions of behaviors ranked for females and males separately. The behaviors we ranked for this analysis are: (1) Infusions during the escalation phase, (2) Front-loading of drug intake in the first 15 min of each self-administration session during the escalation phase, (3) Non-reinforced lever presses during the escalation phase, (4) Short, and (5) Long inter-infusion intervals during the escalation phase, and (6) Escalation of drug intake (day 22 infusions minus day 9 infusions for each rat). To accomplish this, univariate (Pearson’s) correlation analysis was performed separately on male and female data to determine the extent to which each behavioral measure was associated with active lever presses during the reinstatement test (Figures 8A,B; gray highlighted plots). For females, we found significant correlations between reinstatement responding and Infusions, Front-loading, and Long inter-infusion intervals (Figure 8A; R scores listed in each matrix box). For males, we found significant correlations between reinstatement responding and Infusions, Front-loading, Non-reinforced active lever presses, and Short inter-infusion intervals (Figure 8B).

Since many of these behavioral measures were also significantly correlated with each other (Figures 8A,B; unshaded plots), this indicated collinearity of the data. Therefore, to rank the importance of each measure to reinstatement responding in males and in females, we used partial least squares regression, a form of multi-variable linear regression analysis (diagrammed in Figure 8D). The various collinear measures were weighted and combined to generate a single component (“Component,” Figure 8D), which was optimally correlated with post-abstinence reinstatement responding (Figure 8D orange plot, females; Figure 8D blue plot, males). The relative contribution of each measure to active lever presses during the post-abstinence reinstatement test was reported in a sex-specific manner (Figure 8E). Front-loading (average number of infusions during the first 15 mins of each self-administration day during the escalation phase) was most strongly associated with reinstatement responding for both sexes. The behavioral measures with the second highest relative contribution to reinstatement were long-mode inter-infusion intervals for females and short-mode inter-infusion intervals males. The number of oxycodone infusions was ranked third in relative contribution to reinstatement for both females and males. For females, only those three behavioral measures (front-loading, long inter-infusion intervals, and infusion number) were significantly correlated with reinstatement responding, so the relative contributions of other behaviors (Figure 8E, behaviors listed below horizontal dashed line) were deemed insignificant. In contrast, non-reinforced active lever presses also contributed significantly to reinstatement responding in males. Interestingly, escalation itself did not show a significant correlation with reinstatement in either sex (Figures 8A,B; first row, last column).

## Discussion

In this study we found that the majority of addiction-like behavioral components that comprise escalation of oxycodone self-administration and reinstatement of drug-seeking after a 2-week forced abstinence period are similar in male and female Sprague Dawley rats. However, our approach of analyzing more granular components of the process from acquisition of self-administration behavior to patterns of self-administration during the escalation phase and reinstatement showed that the number of non-reinforced active lever presses (those that occur between infusions) was significantly higher in males than females self-administering oxycodone under LgA conditions. In addition, reinstatement responding on the active lever previously paired with oxycodone was significantly higher in females. The second goal of this study was to compute the relative contribution of these granular components of self-administration behavior to the magnitude of reinstatement-responding. We found that many of the same behavioral components contributed the most to reinstatement responding in both males and females, but the rank order of their contribution was sex-dependent. For example, inter-infusion intervals during the escalation phase of self-administration was the second highest contributor for both sexes, except for females the intervals referred to those between bursts of oxycodone infusions and for males the intervals referred to those between individual infusions. We do not think it appropriate to try to directly relate either the behavioral components themselves or the sex differences within to the human condition of OUD or gender differences in OUD. However, we suggest that our findings highlight that broad behaviors (self-administration) and human conditions (OUD) are composed of numerous factors, each of which may reveal sex-dependent mechanistic processes and that it is possible to generate algorithms based on active self-administration (drug-taking) data that can help predict the magnitude of reinstatement to drug-seeking.

In both humans and animal models of addiction-like behavior, it is accepted that the transition from initial drug use to substance use disorder (SUD) involves numerous steps at the behavioral and neurobiological level (Self and Nestler, 1995; Robinson and Berridge, 2001; Wise and Koob, 2014). In this study, we used the extended drug access rodent model of addiction, which is thought to mimic many of these steps (Ahmed and Koob, 1998) because it leads to escalation of drug intake—an essential feature of SUD. Within this construct, we focused our analyses on 6 behavioral measures comprising oxycodone self-administration during the escalation phase (see schematic, Figure 1) that are related to the progression to addiction-like behavior. These included the total number of infusions, front-loading of drug intake, non-reinforced active lever presses, escalation of intake, inter-infusion, and inter-burst intervals. Since relapse to drug-taking is a primary barrier to recovery from SUD, and perhaps OUD in particular (Hunt et al., 1971; O’Brien, 2005), we used reinstatement of oxycodone-seeking after forced abstinence to complete our animal model of addiction-like behavior (Shalev et al., 2002; Venniro et al., 2016). Our experimental design was sensitive to possible sex differences in each of these behavioral components and to reinstatement responding. The fact that sex differences were only observed in non-reinforced active lever presses during oxycodone self-administration and reinstatement responding after abstinence suggests that opioid self-administration as a whole is similar in males and females, but individual steps contributing to reinstatement can be sex-dependent. Although this study was not intended to probe neurobiological mechanisms underlying the behavioral components measured here, it is likely that molecular, hormonal, and pharmacokinetic mechanisms all play a role in male and female behavioral processes contributing to addiction-like behavior. Of note, we have previously shown no sex differences in plasma or brain levels of oxycodone after i.v. infusion (Mavrikaki et al., 2017). It is important to recognize that males and females can show similar outward behaviors that are driven by different underlying mechanisms (Becker and Chartoff, 2019).

In our study, the majority of self-administration behavioral components were similar in males and females. There were no significant sex differences in infusions, active or inactive lever responses during the acquisition or escalation phases (either LgA or ShA conditions). These results are consistent with several recent studies specifically examining male and female rat opioid self-administration behavior under extended access conditions, forced abstinence periods, and reinstatement tests (Venniro et al., 2017, 2019; Fredriksson et al., 2020; Bossert et al., 2022). Even the more granular measures such as front-loading of oxycodone, maintenance of self-administration behavior over the 6-h sessions, and inter-infusions intervals were similar between males and females. In contrast, males, but not females, showed an increase in the number of non-reinforced active lever presses over the course of the escalation phase (days 9–22). These are lever presses that occurred during the ∼10-s period of a drug infusion cycle in which drug was not available. Studies using intermittent access self-administration paradigms typically refer to these non-reinforced active lever presses as “drug-seeking” (O’Neal et al., 2020; Bakhti-Suroosh et al., 2021). However, caution must be used here, as the periods in which drug was not available were much shorter (10 s) than in intermittent access protocols. It is just as likely the increase in non-reinforced active lever pressing reflects oxycodone-induced psychomotor stimulant action or a type of impulsive motor behavior.

Our finding that cue-induced reinstatement is higher in both LgA and ShA females than males after a 14-d forced abstinence period contrasts with the recent studies mentioned above (Venniro et al., 2017, 2019; Fredriksson et al., 2020; Bossert et al., 2022). In those studies, no sex differences in reinstatement were observed. The experimental designs were not identical to ours, as they typically included a reinstatement test after 1 day of abstinence as well as after a prolonged forced abstinence. However, a critical difference is that those studies were conducted during the dark phase of the light cycle (reverse light cycle) whereas ours was conducted during the light phase. It is well known that there are sex differences in the circadian timing system that play important roles in determining responses to both endogenous and exogenous factors (Bailey and Silver, 2014), and it has been shown that time of day/circadian rhythms affect the reinforcing properties of opioids (Webb et al., 2015). We do not claim that circadian rhythms are specifically responsible for our finding that reinstatement responding is higher in females than males, but the “hidden variable” (Butler-Struben et al., 2022) of time of day is likely to be a major factor in the conflicting results emerging in sex difference research. Another factor that likely contributes to conflicting results in studies of sex differences is that estrous cycle can impact drug-seeking behavior (Carroll and Anker, 2010; Becker and Koob, 2016)—although relatively few studies have investigated the effect of estrous cycle on opioid-induced behavior. Indeed, we did not track estrous cycle stages in our studies, as most behavioral endpoints spanned days or weeks, making it practically impossible to tag an estrous cycle stage with a particular behavior.

Our second goal was to determine if the behavioral measures comprising self-administration could be ranked in a sex-dependent manner according to their relative importance to the magnitude of reinstatement-responding. A number of studies report measuring relapse vulnerability and addiction severity by the magnitude of reinstatement responding (Deroche-Gamonet et al., 2004; Belin et al., 2009). For example, it has been proposed that the amount and maintenance of responding for the drug-paired lever during the reinstatement test could predict reinstatement to drug-seeking (Kruzich et al., 1999). But those very behaviors constitute reinstatement and seem not be helpful in a clinical setting. A more useful biomarker would be measurable before relapse occurred, not in retrospect. We first demonstrated that a subset of behavioral measures correlated significantly with reinstatement in males (i.e., infusions, front-loading, non-reinforced active lever presses, and short inter-infusion intervals) and a slightly different subset was found in females (i.e., infusions, front-loading, long inter-infusion intervals). Partial least squares regression analysis then allowed us to rank the relative contribution of each behavior to reinstatement. We found that front-loading behavior, in which rats self-administer more drug at the beginning of each session than at the end, had the highest relative contribution to reinstatement in both sexes. After front-loading, the behavioral measures that ranked the highest for their contribution to reinstatement were sex-dependent. The second ranked contributor was long inter-infusion intervals for females and short inter-infusion intervals for males, followed by number of infusions for both sexes and finally non-reinforced active lever presses for males but not females. The important conclusion from these data is not that we have identified a magic set of factors that “cause” addiction, as the analysis was limited to the measures we input into the analyses. Rather, these findings are significant because they demonstrate that a battery of concrete behavioral measures reflecting active drug-taking behavior can be compared to a human-specific correlation matrix similar to the one we generated in Figure 2 to predict the magnitude, and ultimately the risk, of relapse. Our data also highlight that the predictive behavioral measures and/or their rank order may be sex-dependent, which is consistent with findings that men and women with OUD report different reasons for continued opioid abuse (McHugh et al., 2018).

## Data availability statement

The raw data supporting the conclusions of this article will be made available by the authors, without undue reservation.

## Ethics statement

This animal study was reviewed and approved by McLean Hospital Institutional Animal Care and Use Committee.

## Author contributions

EC conceived the experimental questions and the required analysis methods, wrote the manuscript, and secured funding. SG, YA-C, and JB conceived of the experiments that generated the data used in this study. SG, YA-C, JB, GD, MN, NC, TL, and EK performed experiments. EC, SG, YA-C, and GD analyzed data. All authors contributed to the article and approved the submitted version.
