# Supplementary material for: Ranking the contribution of behavioral measures comprising oxycodone self-administration to reinstatement of drug-seeking in male and female rats
Source: Front Behav Neurosci. 2022 Nov 24;16:1035350. doi: 10.3389/fnbeh.2022.1035350 (PMC9731098; doi:10.3389/fnbeh.2022.1035350)
Supplement: Supplementary file 1 [file Data_Sheet_1.docx]

**Supplementary Methods**

***Procedures specific to rats from Experiment 1***

### Intracranial self-stimulation (ICSS) surgery

Rats were anesthetized with a ketamine/xylazine mixture (ketamine, 80 mg/kg; xylazine 8 mg/kg; IP), and stainless steel monopolar electrodes (0.25-mm diameter; Plastics One, Roanoke, VA) aimed at the medial forebrain bundle at the level of the lateral hypothalamus (2.8 mm posterior to bregma, +1.7 mm lateral to midline, and 7.8 mm below dura) were implanted as described (Carlezon and Chartoff 2007). A non-insulated stainless steel wire was used as the anode and wrapped around a stainless steel screw embedded in the skull. The electrode was fixed in place with three additional skull screws and acrylic dental cement. Rats were allowed to recover from surgery for one week.

### Intracranial self-stimulation (ICSS)

ICSS was performed in standard operant conditioning chambers (ENV-008CT; Med Associates, St. Albans, VT, USA). The chambers were 11.625" L$\times$ 9.78" W$\times$7.35" H. After recovery from surgery, rats were trained to perform ICSS (Carlezon and Chartoff 2007). Using a fixed ratio 1 (FR1) schedule of reinforcement, each quarter turn of a wheel manipulandum earned a 500 millisecond train of square wave cathodal pulses at 158 Hz. The stimulation current was adjusted (final range: 90-200 µA) for each rat to the lowest value that sustained a reliable rate of responding (at least 40 responses per 50 s). Rats were then trained on the 'response rate-frequency' method of ICSS, as in (Ebner et al. 2010; Carlezon and Chartoff 2007). Rats were trained 1 h/d (M-F) for an average of 3–4 weeks until mean ICSS responding remained stable.

### Intravenous self-administration (IVSA) surgery

Once rats had been sufficiently trained on ICSS behavior (2-3 weeks), they were implanted with jugular vein catheters as described in the main methods.

## **Combinatorial IVSA and ICSS**

Combinatorial IVSA and ICSS behavior were used to measure both oxycodone self-administration and reward sensitivity in the same rats. This approach had three segments: (a) ICSS training and determination of baseline ICSS thresholds, (b) 14-d intravenous oxycodone self-administration and post-oxycodone shifts from baseline ICSS thresholds, and (c) 14-d abstinence from oxycodone self-administration and associated shifts from baseline ICSS thresholds (see schematic below). During the combinatorial phase, ICSS (1 h/d) was conducted 2 h after the end of each day’s IVSA session.

**Supplementary Figures**

**Supplemental Figure 1. Examples of oxycodone self-administration behavioral measures from Experiment 1 (IVSA + ICSS rats) and Experiment 2 (IVSA only rats) – presented separately.** For these figures, data are from Experiment 1, in which rats were exposed to ICSS 2-h after each self-administration session and throughout forced abstinence and from Experiment 2, which is a combination of 2 identically run experiments in which rats only performed oxycodone self-administration. (**A, B**) Number of infusions ($\pm$SEM) per IVSA day are shown for males (**A**) and females (**B**) from the long access group (LgA; 6-h/d during escalation phase). Days 1-8 comprise the training phase, and days 9-22 comprise the escalation phase. For males (**A**), there was a main effect of Day (*F*_(2.12, 50.96)_ = 33.45, *p*<0.0001), but no Experiment # x Day interaction or main effect of Experiment #. For females (**B**), there was also a main effect of Day (*F*_(3.56, 88.87)_ = 26.94, *p*<0.0001) but no Experiment # x Day interaction or main effect of Experiment #. These results indicate no significant differences in IVSA behavior between rats that had concurrent IVSA and ICSS and rats that only did IVSA. (**C, D**) Front-loading behavior is shown as number of infusions per 15-min bin in the first hour of self-administration on LgA d9 and LgA d22. For males (**C**), a 3-way ANOVA (Experiment # x Day x Minutes) showed main effects of Minutes (*F*_(1.85, 49.87)_ = 16.58, *p*<0.0001) and Day (*F*_(1.0, 27)_ = 17.08, *p*=0.0003), but no interactions or main effects of Experiment #. For females (**D**), there were main effects of Minutes (*F*_(1.72, 42.93)_ = 25.07, *p*<0.0001) and Day (*F*_(1.0, 25)_ = 20.45, *p*<0.0001), but no interactions or main effects of Experiment #. These results indicate that front-loading behavior is similar in both experiments. (**E**) Reinstatement responding after 14-d of forced abstinence is shown for LgA rats in Experiment 1 (Female, pink bar; Male, green bar) and Experiment 2 (Female, red border; Male, green border). Data are shown as number of presses on the active bar during the 1^st^ hour of the reinstatement test. Similar to combined data presented in the main paper, there was a main effect of Sex (*F*_(1.0, 43)_ = 5.54, *p*=0.023), but no Experiment # x Sex interaction or main effect of Experiment #. N: *Experiment 1*: LgA males, N=13; LgA females, N=11. *Experiment 2*: LgA males, N=13; LgA females, N=16.

**Supplemental Figure 2. Presses on the inactive lever do not change over the course of self-administration.** Data are shown as mean inactive presses per day (1 session/day) for males and females from long access (LgA) **(A)**, short access (ShA) **(B)**, and saline self-administration under LgA conditions **(C)**. *N*: LgA (22 females, 26 males); ShA (15 females, 13 males); Sal (10 females, 9 males).

**Supplemental Figure 3. Male and female rats show escalation of oxycodone intake under both LgA and ShA conditions.** Data are shown as dot and line graphs for Infusions (**A, B, C**) and Active Lever Presses (**D, E, F**) on escalation days 9 and 22 (ESC d9, ESC d22) in male and female rats. Data are analyzed for LgA (**A, D**), ShA (**B, E**), and saline self-administration (**C, F**). For both infusions and active lever presses in LgA and ShA groups, 2-way ANOVA (Sex x Day) showed main effects of Day, with greater responding on ESC d22 compared to ESC d9. **p*<0.05, ***p*<0.01, ****p*<0.001, pairwise comparisons between ESC d22 and d9. N: LgA (22 females, 26 males); ShA (15 females, 13 males); Sal (10 females, 9 males).

**References**

Carlezon, William A., Jr, and Elena H. Chartoff. 2007. “Intracranial Self-Stimulation (ICSS) in Rodents to Study the Neurobiology of Motivation.” *Nature Protocols* 2 (11): 2987–95. https://doi.org/10.1038/nprot.2007.441.

Ebner, S. R., M. F. Roitman, D. N. Potter, A. B. Rachlin, and E. H. Chartoff. 2010. “Depressive-like Effects of the Kappa Opioid Receptor Agonist Salvinorin A Are Associated with Decreased Phasic Dopamine Release in the Nucleus Accumbens.” *Psychopharmacology* 210 (2): 241–52. https://doi.org/10.1007/s00213-010-1836-5.
